# Antioxidants and Antidiabetic Potential of Polyphenolic Fractions and Crude Extracts of *Rhus typhina* Fruit, *Punica granatum* L. Peel, and *Terminalia catappa* L. Leaves: In Vitro and In Vivo Evaluation

**DOI:** 10.1002/cbdv.202500020

**Published:** 2025-03-27

**Authors:** Mudassir Nazir, Muhammad Abdul Haq, Syed Arsalan Ali, Syed Muhammad Ghufran Saeed, Muhammad Ali Ajmal, Muhammad Nisar, Taseer Ahmed Khan, Alexandros Tsoupras, Shahina Naz

**Affiliations:** ^1^ Department of Food Science & Technology University of Karachi Karachi Pakistan; ^2^ Department of Human Nutrition and Dietetics Iqra University Karachi Pakistan; ^3^ Department of Physiology University of Karachi Karachi Pakistan; ^4^ Hephaestus Laboratory, School of Chemistry, Faculty of Sciences Democritus University of Thrace, Kavala University Campus, St Lukas Kavala Greece

**Keywords:** antidiabetic, Indian almond, insulin resistance, polyphenols, pomegranate, sumac

## Abstract

*Rhus typhina* (sumac) fruit, *Punica granatum* (pomegranate) peel, and *Terminalia catappa* (Indian almond) leaves’ extracts and their anthocyanin and non‐anthocyanin fractions were assessed in vitro for 2,2‐diphenyl‐1‐picrylhydrazyl (DPPH) radical scavenging, ferric reducing power (FRAP), human salivary amylase (HAS), and dipeptidyl peptidase IV (DPP‐IV) inhibitory potentials, as well as for their in vivo antidiabetic effects on high‐sugar high‐fat diet (HSHFD) + streptozotocin (STZ) induced diabetic rats (8‐weeks study), by assessing fasting blood sugar, 1 h‐ and 2 h‐oral glucose tolerance tests, serum insulin, homeostatic model assessment (HOMA) analyses, serum creatinine, urea, and blood urea nitrogen. Phytochemical analysis revealed that sumac extract had the highest total phenolic content, total flavonoid content, and total anthocyanin content followed by pomegranate peel and almond leaves. All extracts and fractions showed antioxidant (DPPH and FRAP) and enzyme (HAS and DPP‐IV) inhibition activities and also suppressed STZ effects in diabetic mice by increasing superoxide dismutase, glutathione *S*‐transferase, and insulin, as well as by decreasing HOMA2‐IR, urea, and creatinine, with sumac extract showing benefits even when administered prior to STZ. In addition, in vivo results showed that sumac fruit extract significantly improved glycemic control by reducing fasting blood sugar, enhancing insulin secretion, and improving insulin resistance. These findings suggest that all tested extracts, particularly sumac, possess significant antioxidant, phytochemical, and antidiabetic potential.

## Graphical abstract

This graphical abstract represents the extraction, characterization, and biological evaluation of bioactive compounds derived from specific plant sources. The top section illustrates Rhus coriaria (sumac) fruit, Punica granatum (pomegranate) peel and Terminalia catappa (Indian almond) leaves', which are known for their rich phytochemical composition, including polyphenols, flavonoids, and tannins. These plant materials undergo extraction, depicted by the dropper bottle, to isolate bioactive compounds using suitable solvents and techniques. The extracted compounds are then subjected to in vitro assays for phytochemical profiling and bioactivity evaluation, in vivo studies using animal models to assess pharmacological efficacy, and potential applications in food, nutraceutical, or therapeutic formulations. This study framework emphasizes the scientific approach to exploring plant‐derived compounds for health‐related benefits.

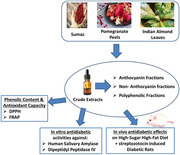

## 1 Introduction

Diabetes or hyperglycemia, a chronic metabolic disorder, affects millions worldwide due to its complications and increasing prevalence. Effective management involves maintaining blood glucose levels, which can be achieved through dietary modifications in mild cases or through pharmacological interventions in more severe cases. Blood glucose originates from glycogen breakdown when needed (non‐fed states) and/or from diet (fed‐state), meaning from several dietary sources like intake of intact glucose and from carbohydrate breakdown by salivary amylase, pancreatic amylase, and disaccharidases, whereas insulin, secreted by pancreatic β cells, and glucokinase regulate its levels. Elevated glucose concentration results from insufficient insulin production (Type 1 diabetes) or ineffective insulin utilization (Type 2 diabetes) [1]. Glucagon‐like peptide‐1 (GLP‐1) and glucose insulinotropic hormone (GIP) promote insulin secretion but are rapidly degraded by dipeptidyl peptidase‐IV (DPP‐IV), suppressing insulin release and elevating glucose levels [2].

Excess glucose activates pathways like the sorbitol pathway, where glucose converts to sorbitol, potentially leading to complications such as retinopathy, nephropathy, and neuropathy [3]. The sorbitol pathway also induces oxidative stress by reducing glutathione reductase activity, contributing to organ damage, including heart disease and cognitive impairment [4]. Diabetic nephropathy, marked by kidney damage, elevated serum creatinine, and blood urea nitrogen, is a severe complication [5].

Diabetes treatments target insulin secretion, action, or inhibition of enzymes like carbohydrolases, DPP‐IV, and aldose reductase. However, synthetic drugs such as α‐glucosidase inhibitors (acarbose, voglibose) and DPP‐IV inhibitors (sitagliptin) have side effects like digestive and respiratory disorders [6]. Natural bioactives that possess medicinal properties, particularly polyphenols, have gained attention due to their low toxicity and multiple mechanisms, including inhibiting carbohydrate‐digesting enzymes, DPP‐IV, and aldose reductase, improving insulin sensitivity, and promoting glucose uptake [7]. For example, phenolic acids and anthocyanins from plant sources like colored potatoes and berries have shown significant antidiabetic potential [8].

On the basis of such reported findings, the rich in bioactive phenolic compounds with well‐known antidiabetic potential, *Rhus typhina*. (sumac), *Punica granatum* L. (pomegranate), and *Terminalia catappa* L. (Indian almond) (Table S1) were selected for the present study. Although their crude extracts exhibit antidiabetic potential, their polyphenolic fractions remain unexplored for their effect on carbohydrases, β‐cell function, and insulin resistance diabetes‐related oxidative stress [9a, 9b]. It has been reported that malic and citric acids in sumac and pomegranate peel inhibit α‐amylase and α‐glucosidase and improve insulin resistance, highlighting their potential role in the activity of polyphenols [10, 11]. Therefore, it is meaningful to find out the influence of these acids on antidiabetic potential of polyphenols in these extracts. Further, the synergistic, additive, or antagonistic effect of organic acids on the antioxidant potential of polyphenols has also been reported which necessitates to rule out the contribution of organic acids in the antioxidant potential of crude extracts [12].

## 2 Results and Discussion

### 2.1 Antioxidant Activity and Bioactive Compounds

**FIGURE 1:**
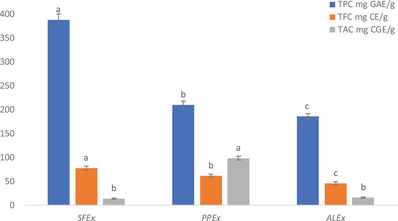
Total phenols (TPC, mg GAE/g), total flavonoids (TFC, mg CE/g), and total anthocyanins (TAC, mg CGE/g) contents in crude extract of sumac (SFEx), pomegranate peel (PPEx), and Indian almond leaves. Values represent mean ± standard deviation. Superscripts among the species are significantly different (*p* < 0.05) as determined by Tukey's multiple test. CE, catechin equivalent; CGE, cyanidin‐3‐glucoside equivalents; GAE, gallic acid equivalent.

The total phenol content (TPC) in sumac fruit extract (SFEx), pomegranate peel extract (PPEx), and almond leaves extract [13] was observed as 388 ± 12.87, 210 ± 8.5, and 185.60 ± 6.33 mg gallic acid equivalent (GAE)/g dry weight (dw) of sample, respectively. On the other hand, total flavonoid content (TFC) was estimated as 77.75 ± 4.27, 61.5 ± 3.33, and 45.6 ± 3.89 mg catechin equivalent (CE)/g dw, whereas total anthocyanin content (TAC) was 14 ± 1.19, 98.56 ± 4.04, and 16 ± 0.95 mg cyanidin‐3‐glucoside equivalents (CGE)/g dw, respectively, in these Exs (Figure 1).

In vitro 2,2‐diphenyl‐1‐picrylhydrazyl (DPPH) radical scavenging activities show that the compounds in extracts/fractions/sumac fruit polyphenolic fraction (Exs/Frs/SFPFr) have the substantial capacity to reduce DPPH radicals. On the basis of IC_50_ values (half maximum inhibitory concentration values), the order of activity was found to be SFEx (13.3µg/mL)>PPEx (18.72µg/mL)>SFPFr (19.23µg/mL)>SFNFr (22.75µg/mL)>PPNFr (23.8µg/mL)>ALEx (26.6µg/mL)>sumac fruit anthocyanin fraction (SFAFr) (27.78µg/mL)>ALNFr (41.4µg/mL)>almond leaves anthocyanin fraction (ALAFr) (48.6µg/mL)>pomegranate peel anthocyanin fraction (PPAFr) (56.22µg/mL) (Table 2). In general, the Exs and non‐anthocyanin fractions (NFrs) showed greater scavenging effect than anthocyanin fractions (AFrs). Concerning species, sumac was found to be the most active, whereas almond leaves were the least active. A strong negative correlation of TPC with DPPH IC_50_ (*R* ^2^ = 0.9534) shows that the higher the TPC, the lower is the amount of Exs/Frs required to inhibit 50% DPPH radicals, which also explains the lowest IC_50_ of SFEx.

The higher antioxidant activity of the crude Exs is based on the high diversity of the constituent compounds and the likelihood of their complex interactions. The antioxidant activity of such complex mixtures is attributed to the synergistic effect of various possible combinations [14]. Compared to AFrs, all NFrs demonstrated higher antioxidant activity (*p* < 0.050) (Table 1). The NFrs contain both flavonoids (including flavanols, flavonols, flavones, and flavanones) and non‐flavonoids (such as tannins and phenolic acids). The important structural features required for a flavonoid to act as a potent antioxidant and free radical scavenger are an o‐diphenolic group in ring B; a 2,3 double bond conjugated with 4‐*oxo* group in ring C and hydroxyl groups in Positions 3 and 5 [15]. Cyanidin‐3‐glucoside, the most abundant anthocyanin in PPAFr (49%) and ALAFr (87%), fulfills all requirements, except the glycoside at position 3, and therefore exhibits considerable DPPH scavenging activity.

**TABLE 1:** Antioxidant capacity of the extracts and fractions derived from sumac fruit, pomegranate peel, and Indian almond leaves, expressed as 2,2‐diphenyl‐1‐picrylhydrazyl (DPPH) radical scavenging activities, ferric reducing power (FRAP) values.

Plant species	DPPH IC_50_ (µg/mL)			
	Exs	AFrs	NFrs	SFPFr
**Sumac fruit**	13.3 ± 1.00^c,4^	27.8 ± 1.0^c,2^	22.7 ± 1.2^b,1^	19.2 ± 1.2^3^
**Pomegranate peel**	18.7 ± 0.7^b,3^	56.2 ± 1.1^a,1^	23.8 ± 1.6^b,2^	
**Indian almond leaves**	26.7 ± 1.3^a,3^	48.6 ± 1.3^b,1^	41.4 ± 0.8^a,2^	

Plant species	FRAP values (mg AAE/g)			
	Exs	AFrs	NFrs	SFPFr
**Sumac fruit**	399 ± 12.0^a,1^	287.5 ± 7.0^a,3^	377.5 ± 5.3^a,2^	381.6 ± 6.1^2^
**Pomegranate peel**	381 ± 7.5^a,1^	133.8 ± 5.0^b,3^	216.0 ± 9.8^b,2^	
**Indian almond leaves**	217.7 ± 10.3^b,1^	138.4 ± 8.57^b,2^	196.5 ± 11^b,1^	

*Note*: Data are presented as mean of triplicate results ± standard deviation. Values with different letters within the same column and with different digits within the same row are significantly different (*p* < 0.05) as determined by Tukey's test.

Abbreviations: AAE, ascorbic acid equivalent; AFrs, anthocyanin fraction; Exs, crude extracts; NFrs, non‐anthocyanin fractions; SFPFr, sumac fruit polyphenolic fraction.

The predominant anthocyanins in SFAFr identified as 7‐methyl‐cyanidin‐3‐galactoside (52.92%), 7‐methyl‐cyanidin‐3‐(2″‐galloyl) galactoside (35.14%), cyaniding‐3‐glucoside (7.84%), and cyanidin‐3‐(2″‐galloyl) galactoside (3.8%) account for more than 99% of the total anthocyanins. Some of these molecules, besides having glycoside at Position 3, also possess galloyl group, which adds three more proton‐donating hydroxyl groups [16]. Thus, the observed higher antioxidant capacity of SFAFr compared to PPAFr and ALAFr (*p* < 0.05) could be explicated by the presence of a galloyl structure in anthocyanins of SFAFr. Likewise, the SFNFr, having gallotannins (penta to decagalloyl‐glucoside) as the dominant group of compounds, was found to be most active among all three NFrs. Yang et al. [17] demonstrated quite higher DPPH scavenging activity of pentagalloyl‐glucoside (IC_50_; 20 µM) in comparison to BHA (44.2 µM). Moreover, Hatano et al. [18] reported an IC_50_ value of 0.66 µM for penta‐*O*‐galloyl‐β‐d‐glucose. Thus, the IC_50_ value of 22.75 µg/mL for SFNFr found in our results could mainly be associated to penta decagalloyl‐glucoside. Pomegranate peel is reported to contain up to 65% punicalagin of total polyphenols, and almond leaves contain about 4.8 mg/g punicalagin as the most abundant tannin [18]. According to Chen et al. [1] punicalagin is one of the major active ingredients to counter reactive oxygen species (ROS) which explains the DPPH scavenging activity of PPNFr (IC_50_; 23.8 µg/mL) and ALNFr (IC_50_; 41.3 µg/mL) found in our results. Strong positive correlation of TFC with DPPH (*R* ^2^ = 0.9949) and weak correlation of TAC with DPPH (*R* ^2^ = 0.1268) indicates less contribution of anthocyanins in DPPH scavenging activity.

The ranking of Exs/Frs with respect to ferric reducing antioxidant power (FRAP) was identical to DPPH (Table 1). The reason for this identical trend is that like DPPH, FRAP reaction is also based on electron transfer to reduce Fe(III) to Fe [19]. Moreover, like DPPH, a strong correlation of TPC and TFC with FRAP (*R* ^2^ = 0.9223 and *R* ^2^ = 0.9999, respectively) and very weak correlation of TAC with FRAP (*R* ^2^ = 0.037) was found. The antioxidant potential of SFPFr was lower (DPPH IC_50_ = 19.23 µg/mL, FRAP = 381.6 µg AAE/g) than SFEx (DPPH IC_50_ = 13.30 µg/mL, FRAP = 399 µg AAE/g), which supports the role of organic acids in increasing the antioxidant capacity of the SFEx. The polyphenols act as efficient hydrogen atom donors for the free radical species. The presence of acids and hence low pH presumably contributes to the slow regeneration of polyphenolic molecules and thus justifies the enhancement of their antioxidant activities [20].

### 2.2 In Vitro α‐Amylase and DPP‐IV Inhibition

All Exs/Frs significantly inhibited HSA with IC_50_ range of 0.16–0.86 mg/mL (Table 2). Except for PPAFr (IC_50_; 0.860 mg/mL) and ALAFr (IC_50_; 0.670 mg/mL), inhibitory activities were comparable to standard inhibitor acarbose (IC_50_ = 0.35 mg/mL). The SFAFr showed greatest inhibition (IC_50_ = 0.16 mg/mL) but not significantly different from SFNFr (IC_50_ = 0.223 mg/mL) and SFEx (IC_50_ = 0.187 mg/mL). The higher anti‐amylase activity of SFAFr relies on >95% of various cyanidin glycosides in this fraction (Table S1).

**TABLE 2:** The IC_50_ values of the extracts and fractions derived from sumac fruit, pomegranate peel and Indian almond leaves against human salivary amylase and DPP‐IV.

	IC_50_ values against human salivary amylase				
Plant species	Exs	AFrs	NFrs	SFPFr	Acarbose
**Sumac fruit**	0.187 ± 0.002^b,3^	0.160 ± 0.026^c,2,3^	0.223 ± 0.025^b,2^	0.372 ± 0.007^1^	0.35 ± 0.03^1^
**Pomegranate peel**	0.362 ± 0.01^a,2^	0.860 ± 0.019^a,1^	0.310 ± 0.026^a,3^		0.35 ± 0.03^2,3^
**Almond leaves**	0.173 ± 0.025^b,3^	0.670 ± 0.025^b,1^	0.178 ± 0.003^c,3^		0.35 ± 0.03^2^

	**IC_50_ values against DPP‐IV**				
**Plant species**	**Exs**	**AFrs**	**NFrs**	**SFPFr**	**Sitagliptin**
**Sumac fruit**	0.175 ± 0.005^b,4^	0.33 ± 0.03^c,2^	0.456 ± 0.025^c,1^	0.244 ± 0.005^3^	0.481 ± 0.017^1^
**Pomegranate peel**	0.376 ± 0.02^a,3^	0.40 ± 0.014^b,3^	0.70 ± 0.026^b,1^		0.481 ± 0.017^2^
**Almond leaves**	0.390 ± 0.01^a,3^	0.48 ± 0.01^a,2^	0.86 ± 0.003^a,1^		0.481 ± 0.017^2^

*Note*: Data presented as the mean of triplicate results ± standard deviation. Values with different letters within the same column and with different digits within the same row are significantly different (*p* < 0.05) as determined by Tukey's test.

Abbreviations: AFrs, anthocyanin fraction; Exs, crude extracts; NFrs, non‐anthocyanin fractions; SFPFr, sumac fruit polyphenolic fraction.

Docking studies of these glycosides with 1SMD showed that all these glycosides (except cyanidin‐3‐glucoside) target the active amino acids of 1SMD (Asp197, Glu233, Asp300) (Figure 2A). 7‐Methyl‐cyanidin‐galactoside comprising 53% of SFAFr and with a binding affinity of −8.2 kcal/mole made four hydrogen bonds (HBs) with Asp197, Glu233, and Asp300, whereas 7‐methyl‐cyanidin‐3‐(2″ galloyl) galactoside (35%, −9.3 kcal/mole) and cyanidin‐3‐(2″ galloyl) galactoside (3.8%, −8.8 kcal/mole) interacted via six and three HBs, respectively, with Asp197, Glu233, and Asp300. Delphinidin‐3‐glucoside, the least (0.25%) of SFAFr, bound the active amino acids via three HBs and one π‐anion bond with Asp 300. It has been demonstrated in many previous studies that the inhibitory effect of flavonoids on α‐amylase depends on their degree of hydroxylation, methylation, and glycosylation [21, 22, 23]. A decrease in the number of free hydroxyl groups due to methylation and glycosylation results in an increase in molecular size, steric hindrance, and polarity, as well as a decrease in the number of hydrogen bond acceptors/donors and a transformation into a nonplanar structure. These changes weaken the binding interaction between flavonoids and α‐amylase [24]. In contrast to this, our docking results show that 7‐methyl‐cyanidin‐3‐(2″galloyl) galactoside has higher binding affinity and made more HBs compared to cyanidin‐3‐(2″galloyl) galactoside. The substitution of ─OH group by methyl group at Position 7 did not affect the binding potential, as ─OH groups in ring B of both compounds interacted with 1SMD. Further, the ligand 7‐methyl‐cyanidin‐galactoside interacted with ISMD through four HBs between the galactose and Asp197, Glu233, and Asp300 which means that glycosylation does not reduce but increases the number of HB acceptors/donors. Both the galloyl glycosides‐7‐methyl‐cyanidin‐3‐(2″galloyl) galactoside and cyanidin‐3‐(2″galloyl) galactoside showed higher binding affinity compared to other cyanidin glycosides due to participation of ─OH in galloyl groups in addition to ─OH in ring B. The inhibitory effect of SFNFr can be explained on the basis of predominance of penta‐ to decagalloyl glucose in SFNFr. Though pentagalloyl glucose (−8.6 kcal/mole) did not interact with any of the active amino acids, it formed 14 HBs and one π‐anion bond with the enzyme which might induce conformational changes and loss of activity. Likewise, sumaflavone (−9.4 kcal/mole), trigallic acid (−8.4 kcal/mole), ellagic acid (−8.2 kcal/mole), and kaempferol (−6.6 kcal/mole) did not target any active amino acid. Only gallic acid (GA) (−6.0 kcal/mole) and quercetin‐3‐glucoside (−8.0 kcal/mole) showed interaction with Asp197, Glu233, and Asp300. Activity‐guided isolation of α‐amylase inhibitory compounds from sumac fruit 37 showed pentagalloyl glucose to exhibit lower IC_50_ value (6.32 ± 0.18 µM) than acarbose (10.69 ± 0.5 µM) against α‐amylase which justifies the HSA inhibition by SFNFr.

FIGURE 2(A) 2D Diagram showing interaction of human salivary amylase (PDB ID 1SMD) with anthocyanin compounds in sumac: (a) 7‐methyl‐cyanidin‐galactoside; (b) 7‐methyl‐cyanidin‐3‐(2″ galloyl) galactoside; and (c) cyanidin‐3‐(2″ galloyl) galactoside. (B) 2D diagram showing interaction of human salivary amylase (PDB ID: 1SMD) with non‐anthocyanin compounds in pomegranate peel and Indian almond leaves: (a) punicalagin; (b) catechin; (c) terflavin; and (d) punicalin. (C) 2D diagram showing interaction of DPPIV (PDB ID: 1NU6) with anthocyanin compounds in sumac fruit: (a) 7‐methyl‐cyanidin‐3‐(2″ galloyl) galactoside; (b) 7‐methyl‐cyanidin‐galactoside; and (c) cyanidin‐3‐glucoside. (D) 2D diagram showing interaction of DPPIV (PDB ID: 1NU6) with anthocyanin compounds: (a) cyanidin‐3,5‐diglucoside; (b) pelargonidin‐3,5‐diglucoside; and (c) pelargonidin‐3‐glucoside. (E) 2D diagram showing interaction of DPPIV (PDB ID: 1NU6) with non‐anthocyanin compounds in Sumac fruit: (a) sumaflavone; (b) trigallic acid; and (c) pentagalloyl glucose. (F) 2D diagram showing interaction of DPPIV (PDB ID: 1NU6) with non‐anthocyanin compounds in Indian almond leaves: (a) punicalin; (b) terflavin; and (c) tergallagin. (G) 2D diagram showing interaction of DPPIV (PDB ID: 1NU6) with non‐anthocyanin compounds in pomegranate peel: (a) catechin; (b) ellagic acid; (c) gallic acid; and (d) punicalin.

**Figure:**
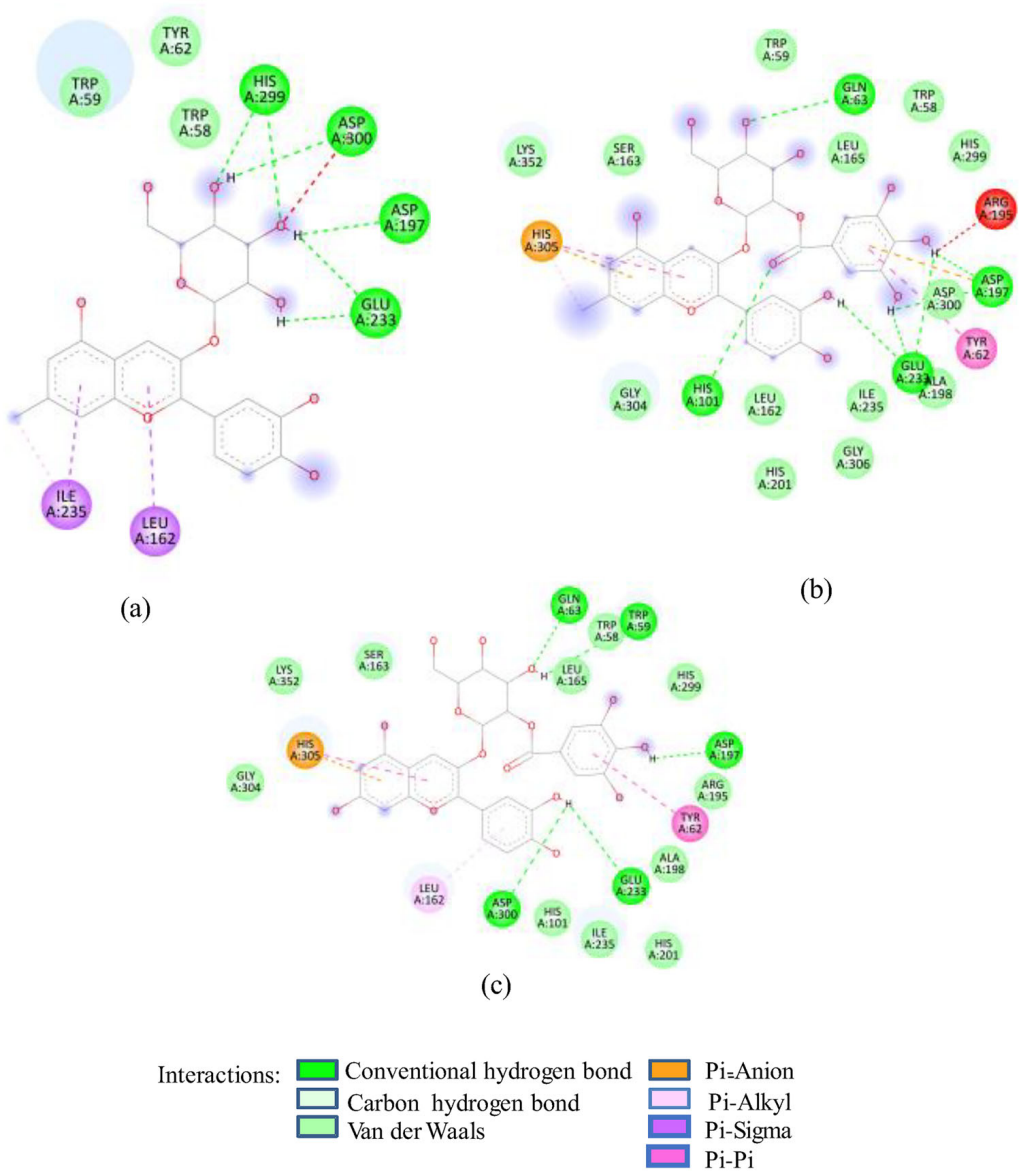

**Figure:**
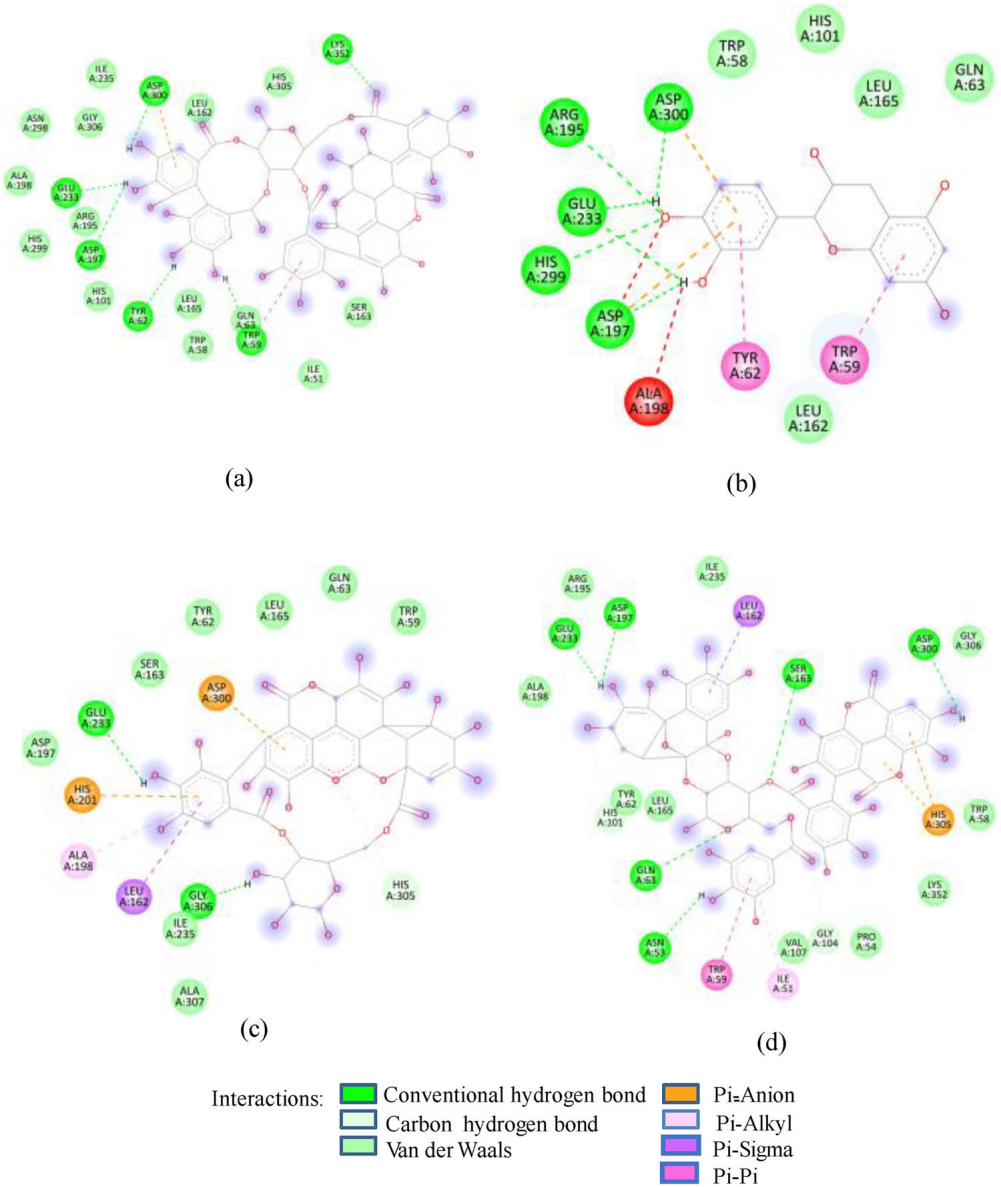

**Figure:**
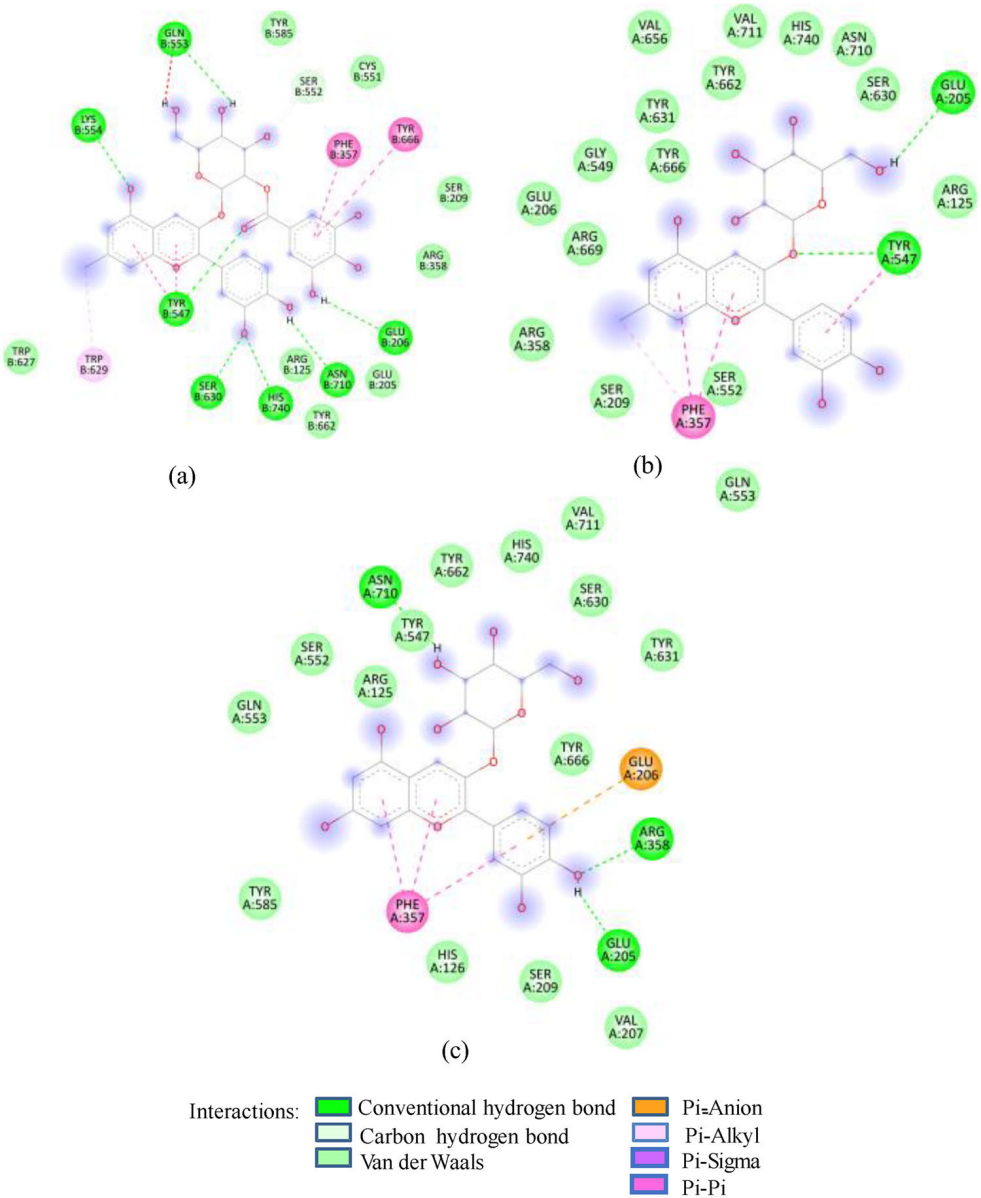

**Figure:**
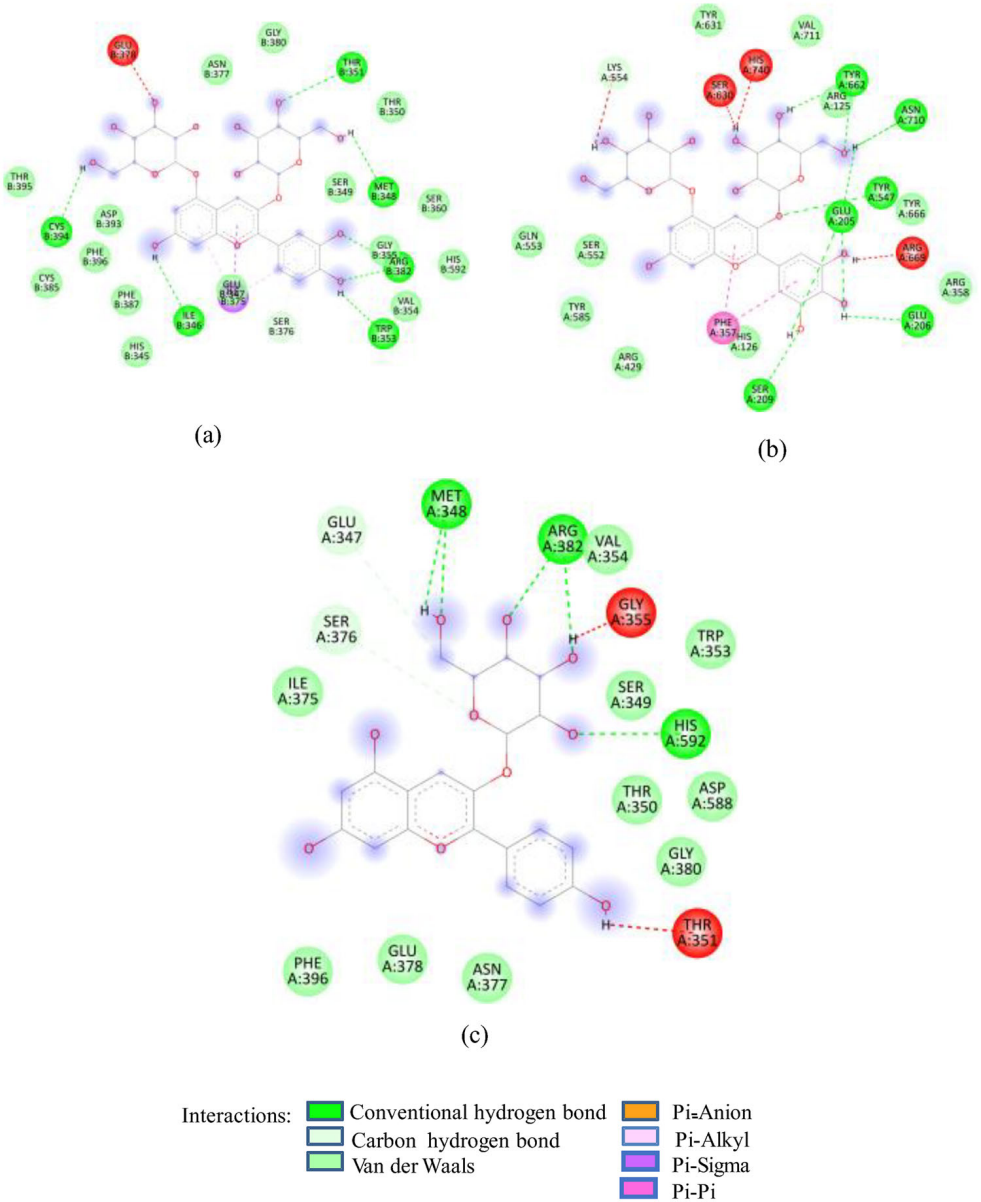

**Figure:**
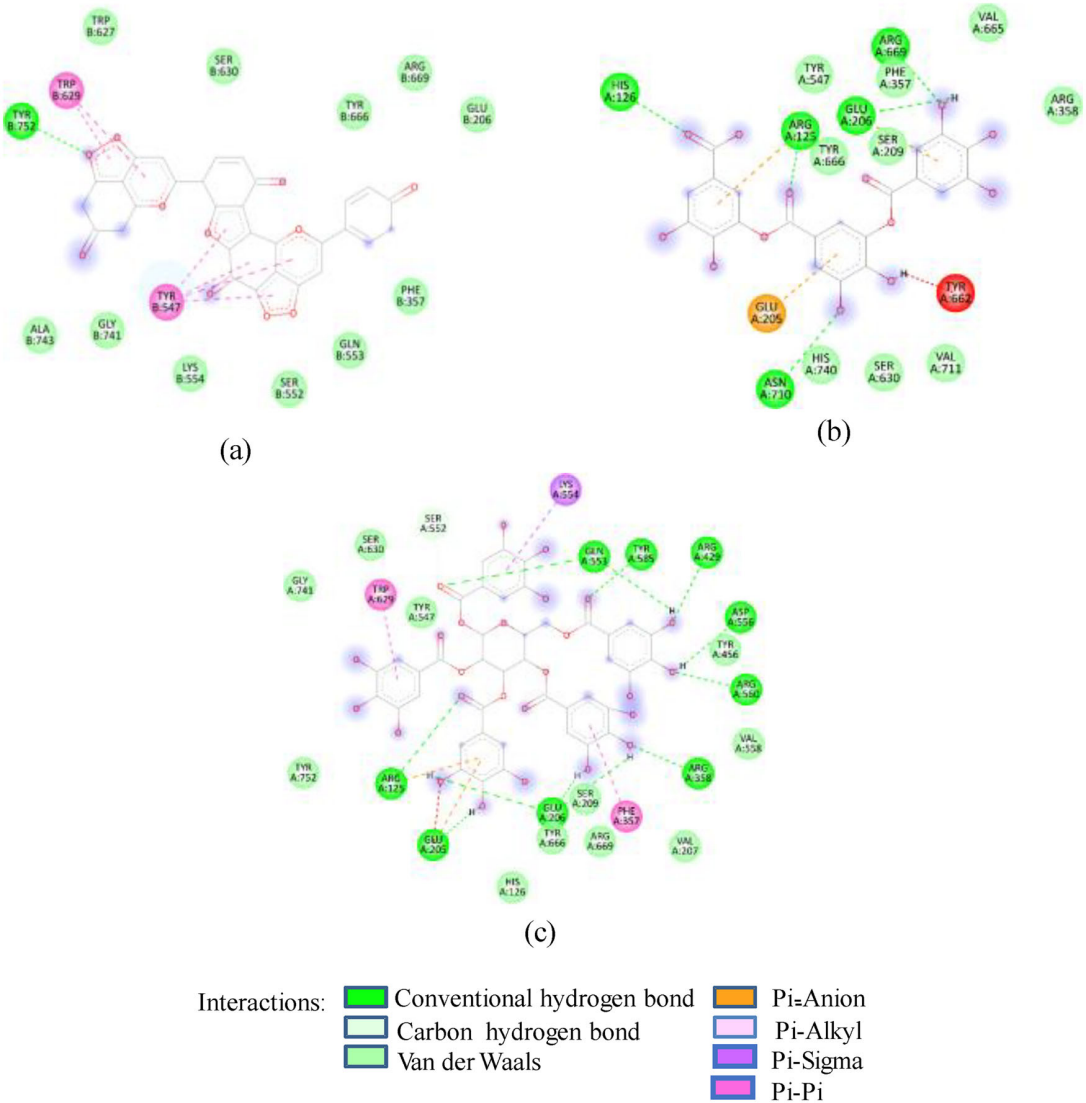

**Figure:**
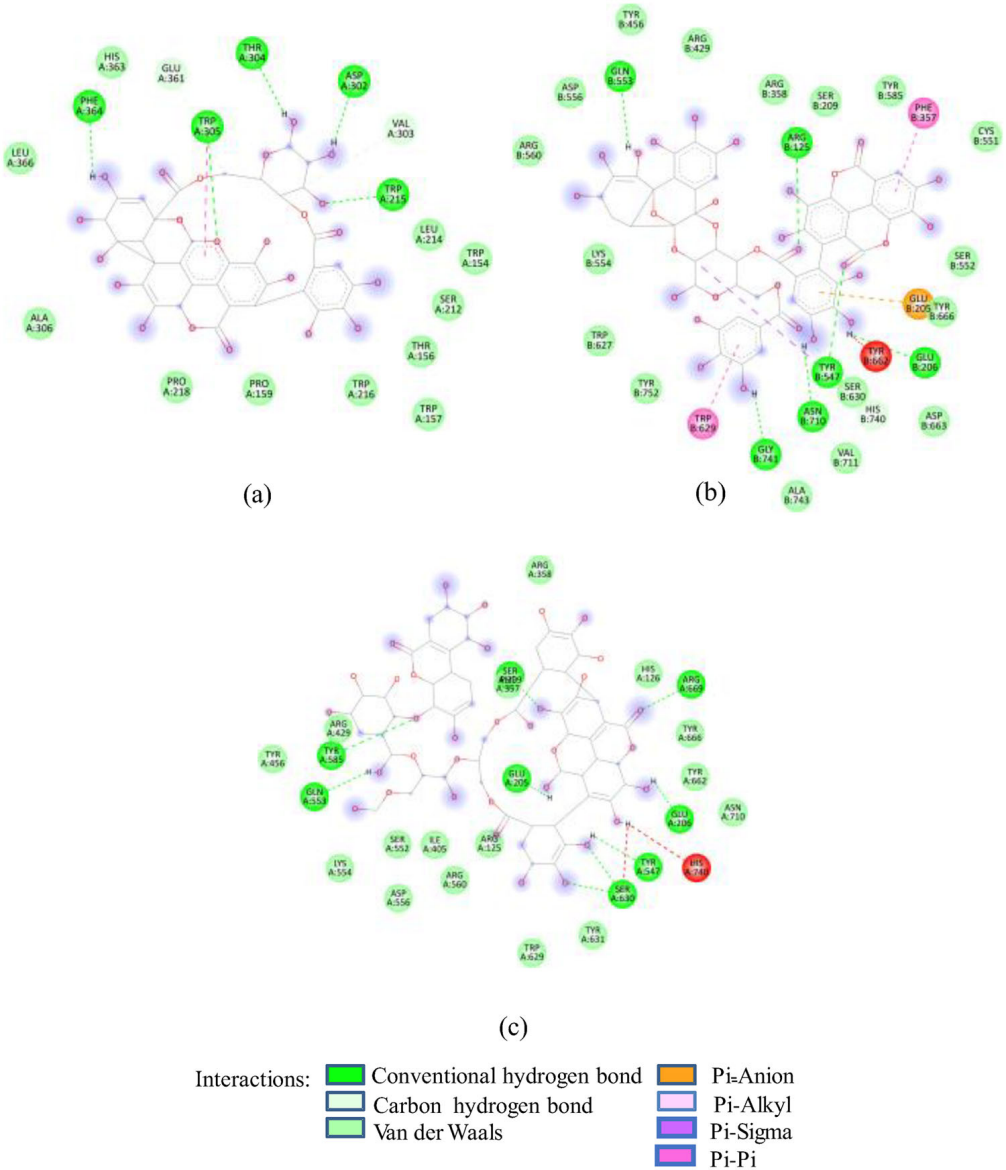

**Figure:**
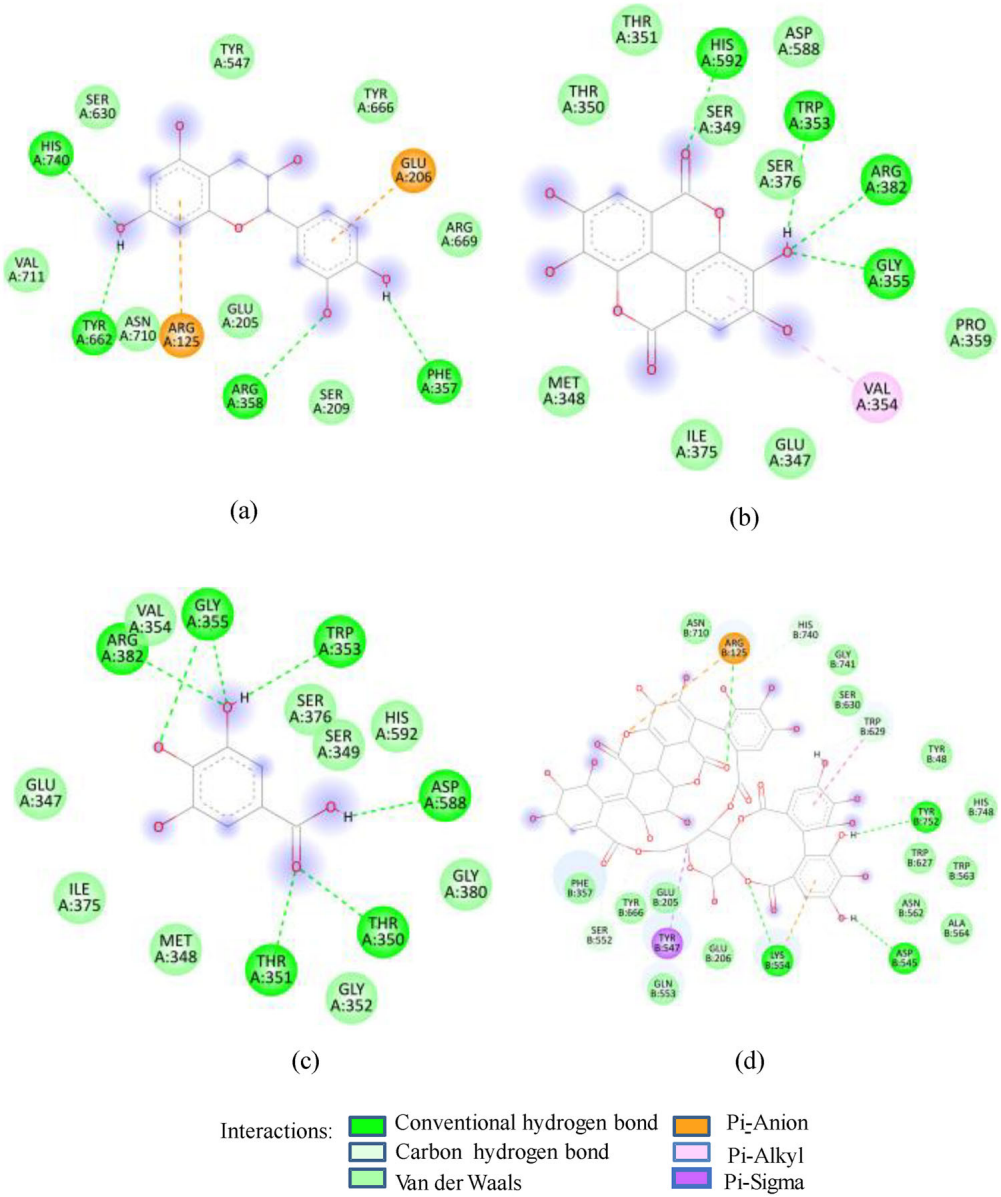

In contrast to SFNFr: PPNFr and ALNFr displayed significantly higher inhibitory potential (IC_50_; 0.31 and 0.178 mg/mL respectively) against HSA compared to their corresponding AFrs (IC_50_ = 0.86 and 0.670 mg/mL). Cyanidin‐3‐glucoside that accounts for 49% and 87% of PPAFr and ALAFr, respectively, does not compete for Asp197, Glu233, and Asp300, whereas punicalagin (−10.1 kcal/mole) which makes 77% of PPNFr and one of the most abundant tannins in ALNFr bound these three amino acids via three HBs and one π‐anion bond. Besides punicalagin, catechin (15% of PPNFr), terflavin (−10.4 kcal/mole) and punicalin (−10.4 kcal/mole) in ALNFr engaged Asp197, Glu233, and Asp300 (Figure 2B). 3,5‐Diglucosides of cyanidin, delphinidin, and pelargonidin exhibited greater binding affinity for 1SMD than their corresponding mono‐glucosides (Table 1) showing increase in affinity due to increase in number of ─OH groups and hence HBs. The IC_50_ of SFPEx (0.372 ± 0.007 mg/mL) was significantly higher than SFEx, SFAFr, and SFNFr (*p* < 0.005) but comparable to acarbose (0.35 ± 0.03 mg/mL). The higher inhibitory effect of SFEx compared to SFPEx establishes the influence of organic acids on the HSA inhibitory effect of SFEx. In digestive enzyme inhibitory assay by Noh et al. [11], citric, malic, and tartaric acids showed dose‐dependent inhibition of α‐amylase. Thus, we can assume that α‐amylase inhibitory effect of SFEx is partly related to citric acid and malic acid in it.

As in the case of HSA, DPP‐IV was strongly inhibited by all Exs/Frs; except SFNFr and ALNFr, IC_50_ values (0.175–0.48 mg/mL) were less than or equal to the standard inhibitor sitagliptin (0.48 mg/mL). The order of activity was SFEx > SFAFr > PPEx > ALEx > PPAFr > SFNFr > ALAFr >PPNFr > ALNFr (Table 2). The amino acid residues that make the catalytic triad of DPPIV are Ser630, Asp708, and His740, whereas the amino acids that are important in substrate recognition and substrate binding include Arg125, His126, Glu205, Glu206, Val207, Ser209, Gly355, Arg356, Phe357, Arg358, Pro359, Glu403, Val404, Ile405, Asp545, Val546, Tyr547, Cys551, Cys552, Gln553, Tyr662, Trp629, Tyr631, Tyr666, Ala707, Asp708, Asp709, Glu738, Asp739, Gly741, Ile742, and Tyr75238. The lower IC_50_ of SFAFr (0.33 mg/mL) compared to PPAFr (0.4 mg/mL) and ALAFr (0.48 mg/mL) is justified on the basis of its anthocyanins which showed high binding affinities and multiple interactions (mainly HBs, π‐π, π‐alkyl, and π‐cation/anion) with these amino acids on docking with DPP‐IV(1NU6). In addition to their phenyl rings and galactose moiety, 7‐methyl‐cyanidin‐3‐(2″ galloyl) galactoside (−9.1 kcal/mole) and cyanidin‐3‐(2″ galloyl) galactoside (−8.7 kcal/mole) engaged galloyl groups to interact with Glu 205, Glu206, Phe357, Tyr547, Cys551, Gln553, Lys554, Tyr662, Trp629, Ser630, and Tyr666 (Figure 2C). 7‐Methyl‐cyanidin‐3‐galactoside (−9.2 kcal/mole), cyanidin‐3‐glucoside (−8.8 kcal/mole), and delphinidin‐3‐glucoside (−8.3 kcal/mole) mostly involved their phenyl rings to bind Arg125, Glu205, Glu206, Phe357, Arg358, Tyr547, Ser552, and Lys554 (Figure 2C). Thus, the cyanidin glycosides and cyanidin galloyl glycosides in SFAFr presumably caused this fraction to inhibit DPPIV strongly. On similar grounds, the inhibitory effect of PPAFr and ALAFr (containing 49% and 87% cyanidin‐3‐glucoside, respectively) could be explained. It has been proved by many researchers that degree of glycosylation in anthocyanins improves their DPP‐IV inhibitory effect 39 but this does not seem to be a consistent rule. Apparently according to 2D interactions diagrams, cyanidin‐3,5‐diglucoside does not interact with any of the active amino acids in 1NU6, whereas cyanidin‐3‐glucoside does; pelargonidin‐3‐glucoside does not target active amino acids, whereas pelargonidin‐3,5‐diglucoside does (Figure 2D). Glycosylation may, however, due to an increase in the number of bonds in general, induce substantial structural changes in the vicinity of catalytic site to discourage substrate binding and hence increase the inhibitory effect. The higher inhibitory activity of SFNFr (0.456 mg/mL) is attributed to the presence of sumaflavone (−10.0 kcal/mole), pentagalloyl glucose (−9.5 kcal/mole), and trigallic acid (−8.6 kcal/mole) which make up most of the SFNFr and target the key amino acids (Arg125, His126, Glu205, Glu206, Ser209, Tyr547, Gln553, Trp629, Arg669, Gly741, and Tyr7520) in 1NU6 pockets (Figure 2E). PPNFr (IC_50_ 0.7 mg/mL) and ALNFr (IC_50_ 0.86 mg/mL) containing punicalagin as the most abundant phenol (77% and 0.48% of dw, respectively) with a high binding affinity (−11.7 kcal/mole) also reduced DPPIV activity by forming HBs, π‐sigma, π‐π, and π‐anion bonds with Arg125, Tyr547, Ser552, Trp629, and Tyr752. Besides punicalagin, catechin (−7.9 kcal/mole), GA (−6.6 kcal/mole), ellagic acid in PPNFr and punicalin (10.4 kcal/mole), terflavin (−10.1 kcal/mole) and tergallagin (−10.9 kcal/mole) in ALNFr contributed to blocking the active site of 1NU6 predominantly through HBs (Figure 2F, G). All three crude Exs exhibited higher inhibitory activity compared to their corresponding Frs, probably because of complex synergistic interactions.

### 2.3 In Vivo Antidiabetic Potential

Following the streptozotocin (STZ) induction, animals from Group 2 (high‐fat, high‐sugar diet [HFHSD]) and Group 3 (HFHSD +150 mg/kg/day *SFEx*) were checked for FBS, 1 h‐oral glucose tolerance test (OGTT) and 2 h‐OGTT on Days 21 and 28. STZ‐induced hyperglycemia in 87% animals of Group 2 (Table 3) with mean FBS, 1 h‐OGTT, 2 h‐OGTT values of 119, 201, and 154 mg/dL, respectively, on Days 21 and 121, 198, and 152 mg/dL, respectively, on Day 28. None of the animals in Group 3 was found diabetic as their mean FBS values were almost close to normal (103 and 101 md/dL) and mean OGTTs were in the normal range (170, 129 mg/dL on Days 21 and 162, 124 mg/dL on Day 28). Nondiabetic control Group 1 was also checked for their FBS and OGTTs for comparison with Groups 2 and 3. All animals in Group 1 remained normal with mean values of 98, 152, 121 mg/dL and 98, 150, 116 for FBS, 1 h‐OGTT, and 2 h‐OGTT, respectively, on Days 21 and 28. The significantly lower values of FBS and OGTTs of Group 3 compared to Group 2 indicate that *SFEx* strongly suppressed the effect of HFHSD and STZ. The mean body weight of rats in Group 2 decreased slightly from an initial value of 198 to 195 g on Day 28, whereas animals in Groups 1 and 3 gained weight up to 215 g and 217 g, respectively (Table 4). This proves the loss in weight due to STZ‐induced diabetes and also the role of *SFEx* in preventing this weight loss.

**TABLE 3:** Effect of high‐fat, high‐sugar diet (HFHSD) with and without crude extract of sumac fruit (*SFEx*) on fasting blood sugar (FBS), 1 h‐oral glucose tolerance test (OGTT) and 2 h‐OGTT values of rats before induction of streptozotocin (STZ) on Days 21 and 28.

Groups with treatments	FBS (mg/dL)		1 h‐OGTT (mg/dL)		2 h‐OGTT (mg/dL)	
	Day 21	Day28	Day 21	Day28	Day 21	Day28
**Group 1 (control)**	98 ± 4.15^b^	98 ± 3.35^b^	152 ± 5.12^c^	150 ± 5.8	121 ± 5.2^c^	116 ± 4.3^c^
**Group 2 (HFHSD)**	119 ± 6.89^a^	121 ± 3.3^a^	201 ± 4.89^a^	198 ± 4.6	154 ± 4.2^a^	152 ± 3.6^a^
**Group 3 (HFHSD + *SFEx*)**	103 ± 4.13^b^	101 ± 4.7^b^	170 ± 3.11^b^	162 ± 5.0	129 ± 4.4^b^	124 ± 4.0^b^

*Note*: Data presented as mean of six values ± standard deviation. Values with different letters within the same column are significantly different (*p* < 0.05) as determined by Tukey's test.

**TABLE 4:** Effect of high‐fat, high‐sugar diet (HFHSD) with and without crude extract of sumac fruit (*SFEx*) on mean body weight of rats before induction of streptozotocin (STZ) on Days 21 and 28.

Groups with treatments	Mean body weight (g)		
	Initial	Day 21	Day 28
Group 1 (control)		206 ± 4.2^a^	215 ± 3.8^a^
Group 2 (HFHSD + STZ)	198 ± 4.5	201 ± 4.3^a^	195 ± 3.5^c^
Group 3 (HFHSD + STZ + *SFEx*)		208 ± 2.8^a^	217 ± 2.5^a^

*Note*: Data presented as mean of six values ±  standard deviation. Values with different letters within the same column are significantly different (*p* < 0.05) as determined by Tukey's test.

Evaluation of the therapeutic effect of *SFEx*, *PPEx*, *ALEx*, *SFPFr* on diabetic rats in comparison with standard drug metformin showed that following intake of glucose, all groups except STZ group were able to regulate blood glucose and achieved glucose homeostasis within 2 h (1 h‐OGTT < 180 mg/dL, 2 h‐OGTT < 140 mg/dL). For STZ group, the values remained higher than normal range till fifth week which infers the role of the *Exs* in controlling hyperglycemia. FBS fluctuated within a narrow but slightly above the normal range (94–109 mg/dL) for Group 2b–2e throughout the study, whereas values were consistently higher for STZ group (134–142 mg/dL) and consistently stable for Group 3 (95–100 mg/dL). Results of metformin group were comparable to Group 3 (Table 5). Following intake of the *Exs* along with the normal diet, the mean body weight of all groups increased but the weight of STZ group remained significantly lower (*p* < 0.05) compared to all other groups which indicates the effect of *Exs* on metabolism and increasing weight (Table 6). Moreover, the weight gain in Group 3 (pretreated with *SFEx*) was comparable to metformin group in Weeks 2–4. As anticipated, administering extracts or metformin to diabetic rats resulted in enhancements in glucose tolerance, glucose‐stimulated insulin secretion, metabolism, and body weight.

**TABLE 5.** In vivo Effects of extracts the derived from sumac fruit (*SFEx*), pomegranate peel (*PPEx*) and almond leaves on fasting blood glucose (FBS), insulin, homeostatic model assessment (HOMA)‐2%B, HOMA‐2%S, HOMA 2‐IR, glutathione‐*S*‐transferase (GST), and superoxide dismutase (SOD) activities of streptozotocin (STZ) induced rats.

Treatment	FBS mg/dL	Insulin µU/mL	HOMA‐2 %B	HOMA‐2 %S	HOMA 2‐IR	GST U/mL	SOD U/mL
**G1: control**	92 ± 2.6^e^	7.9 ± 0.9^d^	93.4 ± 7.2^a^	97.7 ± 12^a^	1.04 ± 0.1^d^	2.3 ± 0.7^b^	7.9 ± 0.8^cd^
**G2a: Met**	102 ± 3.7^cd^	10 ± 1.0^c^	89.0 ± 4.4^a^	74.7 ± 8.2^bc^	1.34 ± 0.14^c^	2.17 ± 0.3^b^	9.5 ± 1.0^bc^
**G2b: *SFEx***	102 ± 5.7^cd^	10.82 ± 1.0^bc^	94.7 ± 8.45^a^	70 ± 7.2^bc^	1.44 ± 0.14^bc^	2.58 ± 0.5^b^	10.3 ± 1.3^b^
**G2c: *PPEx*** **G2d: *ALEx***	110 ± 4.6^b^ 106 ± 7.14^bc^	13.6 ± 0.82^a^ 13.2 ± 1.5^a^	96 ± 10.25^a^ 99.65 ± 11^a^	54.9 ± 3.2^d^ 57.4 ± 6.5^d^	1.82 ± 0.1^a^ 1.76 ± 0.2^a^	1.0 ± 0.6^cd^ 1.25 ± 0.2^c^	5.7 ± 2.0^e^ 7.4 ± 1.0^de^
							
**G2e: *SFPEx***	103 ± 6.7^cd^	11.5 ± 1.0^b^	96.1 ± 12.5^a^	66.7 ± 6.5^c^	1.51 ± 1.5^b^	2.0 ± 0.33^b^	8.6 ± 1.0^bcd^
**G2f: STZ**	140 ± 3.3^a^	<2.9	nd	nd	nd	0.38 ± 0.2^d^	3.7 ± 1.0^f^
**G3: Pre‐*SFEx***	98 ± 2.6^d^	9.76 ± 0.5^c^	94.5 ± 7.2^a^	77.6 ± 3.4^b^	1.29 ± 0.05^c^	4.1 ± 0.9^a^	13.6 ± 3.0^a^

*Note*: Data presented as mean of six values ± standard deviation. Values with different letters within the same column are significantly different (*p* < 0.05) as determined by Tukey's test. nd: values not determined as the insulin level of four out of six animals was less than 2.9 µU/mL.

Abbreviations: G, group; Met, metformin; STZ, streptozotocin.

**TABLE 6:** Effects of extracts derived from sumac fruit (*SFEx*), pomegranate peel (*PPEx*), and almond leaves on body weight of Streptozotocin induced diabetic rats.

Treatment	Mean body weight (g) after STZ Induction				
	Week 1	Week 2	Week 3	Week 4	Week 5
**G1: control**	220 ± 5.8^c^	228 ± 6.0^b^	232 ± 5.7^d^	236 ± 5.3^f^	236 ± 3.8^e^
**G2a: Met**	222 ± 5.0^c^	232 ± 4.8^b^	242 ± 5.6^c^	240 ± 3.6^e^	245 ± 4.3^d^
**G2b: *SFEx***	242 ± 3.4^ab^	245 ± 5.2^c^	248 ± 4.2^b^	248 ± 5.5^cd^	257 ± 6.5^bc^
**G2c: *PPEx***	238 ± 3.2^b^	247 ± 4.38^c^	256 ± 4.0^a^	265 ± 4.0^a^	268 ± 4.0^a^
**G2d: *ALEx***	245 ± 3.06^a^	251 ± 4.8^c^	251 ± 4.3^ab^	256 ± 3.6^b^	261 ± 4.0^b^
**G2e: *SFPEx***	242 ± 3.0^ab^	245 ± 5.2^c^	251 ± 4.2^ab^	253 ± 3.7^bc^	257 ± 6.5^bc^
**G2f: STZ**	205 ± 7.0^d^	199 ± 5.0^d^	193 ± 6.2^e^	195 ± 5.6^g^	191 ± 8.3^f^
**G3: Pre‐*SFEx***	238 ± 4.4^b^	234 ± 3.7^b^	246 ± 2.3^bc^	245 ± 4.0^de^	251 ± 3.5^c^

*Note*: Data presented as mean of six values ±  standard deviation. Values with different letters within the same column are significantly different (*p* < 0.05) as determined by Tukey's test.

Abbreviations: G, group; Met, metformin; STZ, streptozotocin.

Results of FBS, insulin level, and homeostatic model assessment (HOMA) analysis performed after slaughtering at the end of 6 weeks are depicted in Table 5. The serum insulin level of all animals in STZ group was found to be <2.9 µU/mL which indicates severe damage of β‐cells in these animals. Due to this extremely low value, HOMA calculations could not be applied to these animals. Intraperitoneal administration of STZ at a concentration of 40 mg/kg is quite efficient in inducing diabetes in rats [25]. The STZ is known to impair glucose oxidation [26] and cause decline in insulin biosynthesis and secretion [27a, 27b]. It is believed that STZ is taken up by pancreatic β cells via glucose transporter GLUT2. Once inside the β cells, STZ causes alkylation of DNA and results in cell death [28a, 28b] Several other research studies have confirmed that it is the molecule of nitric oxide produced by STZ that is responsible for STZ‐induced DNA damage and destruction of β cells [13, 29] Insulin level in other treated subgroups ranged between 10 and 13.6 µU/mL indicating appropriate production of insulin in animals treated with *Exs*. However, in general, the insulin level was not significantly different among the *Exs* treated subgroups but substantially different from STZ group. The increased level of serum insulin in rats treated with *Exs* suggests that these extracts might stimulate insulin secretion by regenerating and revitalizing β cells.

In vivo antidiabetic effect of sumac has previously been demonstrated, and it has been proved that daily administration of hydroalcoholic extract of sumac (at a dose of 200–300 mg/kg bw for 28 days) in diabetic mice could cause an increase in serum insulin and reduction in blood glucose compared to diabetic control mice [30]. Fatahi Ardakani et al. [31] demonstrated that daily intake of 6 g of sumac powder for 3 months could reduce the insulin resistance in diabetic patients. However, according to our results, 150 mg *SFEx/*kg bw for 6 weeks effectively controlled STZ‐induced diabetes. Likewise, in vivo antidiabetic potential of pomegranate peel and almond leaf extract has also been demonstrated earlier but at much higher dose compared to the dose we used in our experiments. Khalil [32] observed that treatment of diabetic rats with 0.43 g/kg bw of pomegranate peel aqueous extract for 4 weeks decreased blood glucose, increased number of β cells and insulin levels. Iheagwam et al. [9a] showed that aqueous leaf extract of Indian almond at a dose of 400 and 800 mg/kg bw daily for 28 days reversed insulin resistance and improved glucose transport in STZ‐induced diabetic rats. In both of these studies, distilled water was used for extraction from plant material, whereas we used acidified aqueous‐methanol which has been found to be more efficient for the extraction of polyphenols [33]. This might be the reason for the significant therapeutic effect of *PPEx* and *ALEx* at a 150 mg *SFEx*/kg bw in 6 weeks. Though significant differences were found between *SFEx* and *SFPEx* with respect to in vitro antioxidant and anti‐enzymatic activities, no significant difference was found between their in vivo activities. It is believed that low interstitial fluid pH causes insulin resistance via reduction of the binding affinity of insulin to its receptor [12]. Therefore, intake of weak organic acids improves the insulin resistance by elevating the interstitial fluid pH; the carboxyl group of weak acid is absorbed into the body where it behaves as a buffer and elevates the fluid pH. *SFEx*, containing acids besides polyphenols, was expected to cause more decline in insulin resistance compared to *SFPEx* but no noticeable difference between them rules out the contribution of organic acids in lowering the insulin resistance. The least insulin resistance (*p* < 0.05) was shown by the Group 3 treated with *SFEx* for 14 days prior to induction of STZ. HOMA‐IR of this group (1.29) was even lower than the group treated with standard drug metformin (1.34) and quite comparable to nondiabetic control group (1.04) which verifies the protective effect of *SFEx* against STZ (Table 6). STZ caused significant oxidative stress in diabetic rats in 6 weeks as shown by the decrease in superoxide dismutase (SOD) and glutathione‐*S*‐transferase (GST) activities compared to control Group 1 (*p* < 0.05, Table 5). In diabetes, persistent hyperglycemia may cause overproduction of ROS such as O_2_^−^ which can inhibit glycolytic pathway and lead to alternative pathway with accumulation of glucose and production of advanced glycation end products. SOD provides defense against ROS by catalyzing the dismutation of superoxide anion free radical (O_2_^−^) into molecular oxygen and hydrogen peroxide and decreasing O_2_^−^ level [34a, 34b]. Enzyme GST protects cells against reactive metabolites formed from xenobiotics by catalyzing the conjugation of glutathione to xenobiotics, thereby providing protection against oxidative stress. The decrease in GST and SOD in diabetic rats may probably be due to increased utilization of these enzymes to counter the increased formation of ROS on STZ exposure. Oral administration of *Exs* and metformin significantly increased the level of GSH and SOD in diabetic rats. This indicates that the *Exs* either increased the biosynthesis of GST and SOD or helped to reduce oxidative stress [35]. There were no significant differences between the GST and SOD levels in animals treated with different extracts; however, the effect of all extracts was significant compared to STZ group. The increase in GST and SOD in group pretreated with *SFEx* was significantly higher than control and metformin group which shows that antioxidants provided to animals for 14 days prior to the induction of STZ offered enough protection against free radicals and oxidative stress. A high positive correlation was found between total phenolic compounds and GST and SOD (*R* ^2^ = 0.95 and 0.91, respectively); also, the high values of coefficients of correlation for total flavonoids—GST (*R* ^2^ = 0.75) and total flavonoids—SOD (*R* ^2^ = 0.999) suggest that phenolic compounds in *SFEx*, *PPEx*, *ALEx*, and *SFPEx* are mainly responsible for alleviating the oxidative stress induced by STZ in diabetic rats.

It is a very well‐known fact that patients with diabetes are at higher risk of renal decline; therefore, monitoring of their kidney function parameters such as creatinine, urea, and blood urea nitrogen [36] is essential [37]. Serum creatinine, urea, and BUN values of all eight groups were determined to evaluate the impact of STZ‐induced diabetes on kidney function in the presence and absence of *Exs*/metformin (Table 7). Comparison among the different treated groups showed that creatinine, urea, and BUN levels of the STZ group were significantly (*p* < 0.05) higher than groups treated with *Exs*. Except STZ, *PPEx*, and *ALEx* groups, creatinine, urea, and BUN levels of all other groups were comparable to metformin and diabetic control group. Due to the significant impact of STZ‐induced diabetes on renal function, there was a notable increase in both creatinine and urea levels in that group. Treatment with metformin, *SFEx, PPEx, ALEx*, and *SFPFr* reduced serum creatinine and BUN, confirming improvement in the renal functions.

**TABLE 7:** In vivo Effects of extracts derived from sumac fruit (*SFEx*), pomegranate peel (*PPEx*) and almond leaves on urea, creatinine, and blood urea nitrogen values of streptozotocin (STZ) induced rats.

Treatment	Urea (mg/dL)	Creatinine (mg/dL)	BUN (mg/dL)
G1: control	27 ± 4.27^f^	0.6 ± 0.1^d^	12.68 ± 2.2^f^
G2a: Met	33 ± 3.8^def^	0.68 ± 0.27^d^	15.2 ± 5.2^def^
G2b: *SFEx*	34 ± 2.48^de^	0.75 ± 0.15^cd^	15.86 ± 1.2^de^
G2c: *PPEx*	55 ± 6.37^b^	1.6 ± 0.103^b^	25.8 ± 3.28^b^
G2d: *ALEx*	46 ± 6.37^c^	1.06 ± 0.22^c^	21.4 ± 4.51^c^
G2e: *SFPEx*	39 ± 3.5^d^	0.77 ± 0.15^cd^	18.03 ± 3.66^d^
G2f: STZ	62 ± 3.02^a^	2.30 ± 0.62^a^	28.8 ± 1.49^a^
G3: Pre‐*SFEx*	31 ± 7.90^ef^	0.6 ± 0.135^d^	14.55 ± 2.75^ef^

*Note*: Data presented as mean of six values ± standard deviation. Values with different letters within the same column are significantly different (*p* < 0.05) as determined by Tukey's test.

The effect of *SFEx*, *PPEx*, *ALEx*, and *SFPFr* on serum SOD, GST, and creatinine levels could be attributed to the major active compounds in these extracts which have been shown to exhibit antidiabetic effects. Cyanidin‐3‐glucoside at a dose of 4–8 mg/kg bw increased SOD level in ethanol‐induced gastric lesions in rats [38]; cyanidin‐3‐glucoside from black rice prevented renal dysfunction in STZ‐diabetic rats [39]. Similarly, punicalagin and pentagalloyl glucose have been shown to cause increase in SOD activity [40] and decrease in creatinine levels [41a, 41b] in diabetic rats.

## 3 Conclusions

The extracts of *R. typhina* (sumac) fruit, *P. granatum* (pomegranate) peel, and *T. catappa* (Indian almond) leaves demonstrated multifunctional antidiabetic potential. These extracts effectively inhibited salivary amylase, pancreatic amylase, and DPP‐IV in vitro and improved glycemic control in vivo by maintaining normoglycemia, enhancing β‐cell function, and reducing insulin resistance. Furthermore, the extracts demonstrated strong antioxidant activity, reducing oxidative stress and enhancing renal function indicators, including creatinine and urea levels in diabetic rats. When given before diabetes induction, the sumac extract showed the highest activity among the studied extracts, including a protective effect. These results offer compelling evidence for additional investigation into these plant extracts as possible therapeutic agents for the treatment of diabetes and its consequences. In addition, future studies should focus on isolating active polyphenols, exploring their mechanisms in insulin signaling, and conducting long‐term in vivo studies. Clinical trials are needed to validate their efficacy, and their potential for functional food or nutraceutical development should be explored.

## 4 Experimental Section

This study fractionated crude methanolic extracts of sumac fruit, pomegranate peel, and almond leaves using HLB‐SPE cartridges into AFrs and NFrs. A SFPFr of sumac was also prepared by removing non‐polyphenolic components. The antioxidant activity and inhibitory effects on human salivary amylase (HAS) and DPP‐IV were assessed in vitro, whereas in vivo therapeutic effects on STZ‐induced diabetic rats were evaluated. To isolate the impact of organic acids, SFPFr was tested separately. Additionally, SFEx was administered preemptively to demonstrate its protective effect. Molecular docking of major phenolic compounds (isolated and identified previously in sumac fruit, pomegranate peel, and almond leaves) (Table S1) was performed with HAS and DPP‐IV to compare their binding energies and inhibition potential with standard drugs acarbose and sitagliptin.

### 4.1 Collection of Raw Materials

The Indian almond leaves were collected from the nursery of the University of Karachi (UOK), Karachi, Pakistan, in November 2023. The sumac fruits and pomegranate peels were procured from the local market of Northern areas of Pakistan in November 2023. The plant materials were identified and authenticated by Dr. Muneeba Khan, a taxonomist at the Herbarium, UOK. Voucher specimens were deposited at the Botanical Garden Herbarium, UOK, as follows: *R. typhina* (S. No. 448), *P. granatum* (Voucher No. 99776), and *T. catappa* (Voucher No. 99774). All plant samples were collected during their optimal maturity stage. The samples were sorted, washed, packed, and stored at −15°C.

[Correction added on May 23 2025, after first online publication. The Voucher No. was replaced with “S. No. 448” in this version]

### 4.2 Chemicals and Reagents

All solvents and chemicals were analytical grade and purchased from either BDH or Merck. HLB columns (Oasis, USA) were used for fractionation of polyphenols. 2,2‐Diphenyl‐1‐picrylhydrazyl (DPPH) radical, HAS (A1031), DPPIV (D4943), and STZ were purchased from Sigma‐Aldrich, Germany.

### 4.3 Extraction and Fractionation of Polyphenols

All plant samples were extracted with acidified methanol (85:15 (v/v), where 85% methanol was mixed with 15% acid‐0.01 N HCl). The extracts (*Exs*) were concentrated at low temperature on rotary evaporator (Rotavapor R‐300, Büchi Labortechnik AG, Flawil, Switzerland) under vacuum and freeze‐dried by using freeze dryer (FreeZone 6 Liter Benchtop Freeze Dryer, Labconco Corporation, Kansas City, MO, USA). To obtain *SFPFr*, *AFrs*, and *NFrs*, the procedure of Kim and Lee [42] was used with some modifications. The crude acidified methanolic extracts were first adsorbed on hydrophilic–lipophilic balanced (HBL) solid‐phase extraction column (Oasis HLB, 6 cm^3^, 200 mg, Waters Corporation, Milford, MA, USA) preconditioned sequentially with ethyl acetate, absolute methanol, and 0.01 N aqueous HCl. The columns were subsequently washed with 0.01 N aqueous HCl to remove sugars and acids and dried. For *SFPFr*, dried column was directly eluted with acidic methanol, whereas in order to obtain fractions, columns were first washed with ethyl acetate to elute *NFrs* and then acidic methanol to elute *AFrs*. The solvents ethyl acetate and methanol were removed from samples under reduced pressure at 30°C, and the fractions were stored at −4°C.

### 4.4 Total Phenolic Contents

The TPC was determined by using Folin–Ciocalteu assay [43]. The sample (300 µL) was mixed with 160 µL Folin–Ciocalteu reagent and 250 µL of distilled water. After 5 min, 300 µL of 10% sodium carbonate solution was added, and the reaction mixture was incubated for 30 min at room temperature. The absorbance was measured at 750 nm using a UV–visible spectrophotometer (Agilent Technologies, Agilent 8453, Santa Clara, CA, USA). The results were expressed as mg of GAE [44]/g sample.

### 4.5 Total Flavonoid Contents

The TFC in each sample was measured by the aluminum chloride colorimetric method using catechin as the standard [45]. To 100 µL of sample, 30 µL of 5% sodium nitrite and 400 µL of distilled water were added. After 5 min, 300 µL of 10% aluminum chloride was added, and the reaction mixtures were held for 6 min. Then 200 µL of 1 M sodium hydroxide and 240 µL of distilled water were added, and the absorbance of the solution was measured immediately at 510 nm. TFC was expressed as mg of CE/g sample.

### 4.6 Total Anthocyanin Contents

The pH differential method determined the TACs of the samples [46]. An adequate quantity of samples was mixed separately with 0.025 M hydrochloric acid–potassium chloride buffer (pH = 1) and 0.4 M sodium acetate buffer (pH = 4.5) to have an absorbance reading between 0.2 and 1.4. The absorbance of each sample was measured at 520 and 700 nm, and the TACs were expressed as CGE/g sample:

(1) $$\;\mathrm{Anthocyanin}\;\mathrm{pigment}\;\left(\mathrm{mg}/L\right)=\frac{A\times \;\mathrm{MW}\;\times \;\mathrm{DF}\;\times \;V\;\times \;1000}{a\;\times l\;\times m}$$

where *A* is the absorbance, MW is the molecular weight of cyanidin‐3‐glucoside (449.2 g/mol), DF is the dilution factor, V is the solvent volume (mL), *a* is the molar absorptivity (26 900 L/mol/cm), and *l* is the cell path length (1 cm).

### 4.7 Antioxidant Activity

#### 4.7.1 DPPH Scavenging Method

To determine the effect of *Exs, Frs*, and *SFPFr* on DPPH radical, 1 mL (100 µM) solution of DPPH in methanol was mixed with 200 µL of sample containing 0.2, 0.05, 0.025, 0.0125, and 0.00625 mg. The mixtures were shaken vigorously, left in the dark at room temperature for 20 min, and then their absorbance was measured at 517 nm [47]. The control was prepared by mixing all the reagents except sample. The DPPH scavenging capacity was calculated using the following equation:

(2) $$\mathrm{Scavenging}\;\mathrm{activity}\;\left(\%\right)=\frac{A_{0}\;\times A_{s}}{A_{0}}\times 100$$

where *A*_0_ is the absorbance of control, and *A_s_* is absorbance in the presence of the sample. The results were plotted as the % scavenging activity against the concentration. The IC_50_ values were expressed as GAE [44] required for 50% of free radical scavenging activity. The GA was used as a positive control.

### 4.8 FRAP Method

The method described by Benzie and Strain [48] was followed for the determination of FRAP. The FRAP reagent (2.3 mL) was mixed with 0.7 mL of samples. The mixture was then incubated at 37°C for 30 min in the dark. The absorbance was measured at 593 nm against a blank having all the reagents excluding the sample. Ascorbic acid was used as the standard, and results were expressed in µg of ascorbic acid equivalents per gram (µg AAE)/g.

### 4.9 In Vitro Enzyme Inhibition Assays

#### 4.9.1 α‐Amylase Inhibition

The α‐amylase inhibition of samples was evaluated by following the procedure of Unuofin et al. [49] with modifications. The HAS (0.3 mL, 0.8 U/mL) was mixed with 0.3 mL sample and 0.6 mL of phosphate buffer (pH 6.9). The mixture was incubated at 37°C for 15 min. Then 0.4 mL of this incubated mixture was transferred to test tubes containing 3 mL of 1% starch solution and 2 mL phosphate buffer. The mixtures were incubated again for 45 min. At zero time and after 45 min, 0.1 mL of the reaction mixture was taken and added to 10 mL iodine solution (0.245 g *I*_2_ + 0.4 g KI in one liter), and its absorbance was recorded at 565 nm. Acarbose (Sigma‐Aldrich, St. Louis, MO, USA) was used as positive control, and % inhibition was calculated as

(3) $$\begin{array}{ccc} & & \%\;\alpha \;\mathrm{amylase}\;\mathrm{Inhibition}\\ & & =\frac{\left(A_{0}-A_{t}\right)\mathrm{control}\;-\left(A_{0}-A_{t}\right)\mathrm{sample}}{\left(A_{0}-A_{t}\right)\mathrm{control}}\times 100\; \end{array}$$

where *A*_0_ is absorbance at 0 time, and *A_t_* is absorbance at 45 min.

### 4.10 DPP‐IV Inhibitory Activity

The DPP‐IV inhibitory activities of the samples were determined according to the method of Nongonierma et al. [50]. Briefly, 50 µL of sample and 50 µL of 0.8 mmol/L substrate (Gly‐Pro‐*p*‐nitroanilide, G0513, prepared in 100 mmol/L Tris–HCl, pH 8 buffer) were mixed, and this mixture was pre‐incubated at 37°C in a thermomixer at 1000 rpm for 10 min. To initiate the reaction, 100 µL of DPP‐IV (0.01 U/L) was added to the mixture, and after 1 h, the reaction was stopped by adding 200 µL of 1 mol/L sodium acetate (pH 4.0) buffer. The concentration of *p*‐nitroanilide (*p*‐Na) so formed was determined spectrophotometrically at 405 nm. Percent inhibition values for the samples were calculated in comparison to negative controls. In the sample control group, buffer replaced the sample solution, whereas in the blank control group, the enzyme solution was replaced by buffer. Sitagliptin (Sigma‐Aldrich, St. Louis, MO, USA) was used as reference drug:

(4) $$\begin{array}{ccc} & & \%\;\mathrm{DPP}-\mathrm{IV}\;\mathrm{Inhibition}\\ & & =\frac{\left(A\;\mathrm{control}-\;A\;\mathrm{blank}\;\mathrm{control}\right)-\;\left(A\;\mathrm{sample}\;-\;A\;\mathrm{sample}\;\mathrm{control}\right)}{\left(A\;\mathrm{control}\;-\;A\;\mathrm{blank}\;\mathrm{control}\right)}\\ & & \times \;100 \end{array}$$

where *A* is absorbance. The IC_50_ values were obtained by plotting mean %inhibition against concentration of sample.

### 4.11 In Vivo Effect on STZ‐Induced Diabetic Rats

#### 4.11.1 Animals and Ethics

A total of 70 healthy male rats weighing 200 ± 10 g (purchased from International Center for Chemical and Biological Sciences (ICCBS), UOK) were used for this study. Female rats exhibit difficulty in developing diabetes on a high‐fat diet compared to males. Consequently, only male rats were selected for the study. The animals were acclimatized to lab conditions for 7 days prior to the start of the study. CCTV cameras were used to monitor the activities and responses of rats throughout the study. The research was conducted in accordance with internationally recognized principles for the use and care of laboratory animals as outlined in the NIH Guide for the Care and Use of Laboratory Animals (Publication No. 8023, revised 1978), under the oversight and approval of the Institutional Bioethical Committee at the University of Karachi, Pakistan (Approval number: IBC KU‐379/2023). Animals were kept (in groups of three) in well‐ventilated polypropylene plastic cages at the animal house where temperature was maintained at 22°C, relative humidity around 50% under regulated light (12 h day/night cycle). HFHSD (D12451 45% calories from fat) was given to all groups for 14 days (except control group which was given normal standard pelletized feed) prior to induction of STZ with free access to drinking water to all.

### 4.12 Experimental Design and Animal Treatments

The therapeutic efficacy of *SFEx, PPEx, ALEx*, and *SFPFr* in STZ‐induced diabetic rats was assessed by comparing their effects with the standard drug metformin. Additionally, we investigated the preventive potential of *SFEx* by administering it to animals prior to the induction of diabetes. Initially, the rats were divided into three main groups (Table S2).

On the 15th day, Groups 2 and 3 were injected intraperitoneally with 40 mg/kg STZ (dissolved in sodium citrate buffer, pH 4.5) to induce diabetes. After STZ induction, OGTTs and body weight measurements were conducted on Days 21 and 28. The 87% of rats (40) that were found diabetic with 2 h‐postprandial blood glucose (2 h‐PBG) level of >140 mg/dL [45] were selected from Group 2 and divided into six subgroups (*n* = 6 for each group) for six different treatments to be given with normal standard diet for 6 weeks (Table S3).

Although none of the animals in Group 3 were diabetic, 6 animals were selected and continued to be fed with normal diet + 150 mg/kg/day *SFEx* for 6 weeks. The body weight and OGTT of all animals groups were checked every week till the fifth week. At the end of 6 weeks, rats were sacrificed humanely after 16 h of fasting by following institutional guidelines. Euthanasia was performed under deep anesthesia (intraperitoneal injection of sodium pentobarbital (60 mg/kg) to ensure minimal distress to the animals. The blood samples were collected and centrifuged at 4000 rpm for 5 min to separate serum for measurement of insulin, GST, SOD, urea, and creatinine.

### 4.13 OGTT Assay

The rats were orally given 2 g/kg glucose after 16 h of fasting for OGTT. The blood samples were collected from the tail vein, and the blood glucose levels were measured at 0, 60, and 120 min using a glucometer (Accu‐Chek, Roche, USA).

### 4.14 Serum Insulin, GST, and SOD Activities

The serum insulin, GST, and SOD were measured using enzyme linked immunosorbent assay [51] kits (Catalog no: MBS045315, E1989Ra, E1444Ra, respectively, from MyBioSource San Diego, CA, USA, and Bioassay Technology Laboratory, Shanghai, China).

### 4.15 Serum Creatinine and Urea

Serum creatinine was determined by the Jaffe colorimetric method (Catalog no: STA378, USA), whereas urea kit method was based on the Berthelot reaction (Catalog no: STA 382, USA). Blood urea nitrogen [36] was calculated using the following formula:

(5) $$\mathrm{Urea}\left(\mathrm{mg}/\mathrm{dL}\right)\times \;0.467=\mathrm{BUN}\left(\mathrm{mg}/\mathrm{dL}\right).$$

### 4.16 Docking in AutoDock Vina

All selected ligands were docked with the receptor proteins using the virtual screening tool of PyRx [52] which uses AutoDock Vina (version 1.1.2, The Scripps Research Institute, La Jolla, CA, USA) as a docking software to generate input files, Open Babel (Version 3.1.1) for importing SDF (Structure Data File) and Python (Version 3.x, Python Software Foundation) as a programming language. Among the nine conformations of each ligand obtained as a result of autodocking, the one with maximum binding affinity was taken into consideration. Discovery Studio (Version 20.1, Dassault Systèmes, Vélizy‐Villacoublay, France) was used for post‐docking analysis and detailed ligand–receptor interactions in 2D and 3D formats.

### 4.17 Ligand Selection and Preparation

The major polyphenolic compounds identified previously in *SFEx*, *PPEx*, and *ALEx* were selected for docking (Table 1). Their structures were downloaded from the PubChem website and converted to PDB format using PyMOL (Version 2.4.0, Schrödinger, LLC, New York, NY, USA). For docking in AutoDock Vina (Version 1.1.2, The Scripps Research Institute, La Jolla, CA, USA), all the structures were first opened in Open Babel to optimize the geometry and minimize the energy and then saved in Protein Data Bank, Partial Charges, & Torsions (PDBQT) format.

### 4.18 Protein Preparation

The 3D structures of the enzymes, salivary amylase and DPP‐IV, were obtained in PDB format from the Protein Data Bank with PDB IDs as mentioned in Table S4. For docking in AutoDock Vina, structures were loaded in the software to create a PDBQT file containing protein structure with hydrogens in all polar residues and were saved in the directory of macromolecules in PDBQT format. The docking site on protein target was defined by a grid box, the grid centers *x y z* coordinates, and the grid box size for each protein was set as mentioned in Table 4. Grid box dimensions were maximized to cover the whole macromolecule and to search for all the probable binding sites available on the surfaces.

### 4.19 Statistical Analysis

All the experiments were conducted in triplicate, and results were expressed as mean ± standard deviation. Data were analyzed statistically by Statistical Package for Social Software Design (SPSS, Version USA) by applying Tukey's test to measure differences between pairs of means at *p* < 0.05. The molecular docking was performed using AutoDock Vina (version 1.1.2, The Scripps Research Institute, La Jolla, CA, USA). The virtual screening tool PyRx was used to generate input files, whereas Open Babel (version 3.1.1) was employed for structure conversion. Discovery Studio (version 20.1, Dassault Systèmes, France) was utilized for post‐docking analysis, providing detailed 2D and 3D ligand–receptor interactions.

## Author Contributions

**Mudassir Nazir, Muhammad Abdul Haq, Syed Arsalan Ali, Shahina Naz and Alexandros Tsoupras**: writing original draft writing original reviewing writing original editing, writing original analysis. **Shahina Naz, Muhammad Abdul Haq and Syed Muhammad Ghufran Saeed**: supervising, administrating, software, and conceptualizing. **Muhammad Ali Ajmal, Muhammad Nisar and Taseer Ahmed Khan**: experimentation.

## Ethics Statement

The research was conducted by internationally recognized principles for the use and care of laboratory animals as outlined in the NIH Guide for the Care and Use of Laboratory Animals (Publication No. 8023, revised 1978), under the oversight and approval of the Institutional Bioethical Committee at the University of Karachi, Pakistan (Approval number: IBC KU‐379/2023).

## Conflicts of Interest

The authors declare no conflicts of interest.

## Supporting information



Supporting Information for this article is available on the WWW under https://doi.org/10.1002/MS‐number.

## Data Availability

The data that support the findings of this study are available from the corresponding author upon reasonable request.
