# Supplementary material for: Antioxidants and Antidiabetic Potential of Polyphenolic Fractions and Crude Extracts of Rhus typhina Fruit, Punica granatum L. Peel, and Terminalia catappa L. Leaves: In Vitro and In Vivo Evaluation
Source: Chem Biodivers. 2025 Mar 27;22(7):e202500020. doi: 10.1002/cbdv.202500020 (PMC12270376; doi:10.1002/cbdv.202500020)
Supplement: Supplementary file 1 — Supporting Information for this article is available on the WWW under https://doi.org/10.1002/MS‐number. [file CBDV-22-e202500020-s001.docx]

**Supplementary Table 1:** Phenolic compounds identified in sumac fruit, pomegranate peel and Indian almond leaves

| **Anthocyanin** | **Non- Anthocyanins** |
| --- | --- |
| Sumac [^15^](#_ENREF_18) | |
| 7-Methyl-cyanidin-3-galactoside 53%^a^  7-Methyl-cyanidin-3-(2״galloyl) galactoside 35%^a^  Cyanidin-3-glucoside 7.8%^a^  Cyanidin-3- (2״galloyl) galactoside 3.8%^a^  Delphinidin-3-glucoside 0.25%^a^ | Sumaflavone^qnd^  Pentagalloyl glucose  Trigallic acid^qnd^  Gallic acid^qnd^  Ellagic acid^qnd^  Kaempferol^qnd^  Quercetin^qnd^ |
| Pomegranate peel [^15-16^](#_ENREF_18) | |
| Cyanidin-3-glucoside 49%^a^  Pelargonidin-3-glucoside 25%^a^  Cyanidin-3,5-diglucoside 12%^a^  Pelargonidin-3,5-diglucoside 6%^a^  Delphinidin-3-glucoside 5%^a^  Delphinidin-3,5-diglucoside 3%^a^ | Punicalagin 77%^b^  Catechin 15%^b^  Ellagic acid 3%^b^  Gallic acid 3%^b^ |
| Indian almond leaves [^17^](#_ENREF_20) | |
| Cyanidin-3-glucoside 87%^a^ | Punicalagin (ca. 0.48% of the dry weight)  Punicalin^qnd^  Tergallagin^qnd^  Terfalvin^qnd^ |

^a^: % of total anthocyanin content; ^b^: % of total monophenols (monophenols =76% of total phenols); ^qnd^: major compounds but quantity not determined.

**Supplementary Table 2:** Initial grouping of animals

| Groups | Group Description | Number of Animals (n) | Diet/Treatment |
| --- | --- | --- | --- |
| Group 1 | Non-diabetic, control group | 10 | Normal feed throughout |
| Group 2 | Diabetic (treated) group | 50 | High-fat high-sugar diet (HFHSD) |
| Group 3 | Pretreatment group | 10 | HFHSD + 150 mg/kg/day *SFEx* for 14 days |

**Supplementary Table 3:** Group division for Treatment (Group 2 Diabetic Rats)

| Group ID | Subgroup Description | Treatment | Dose | Treatment Duration |
| --- | --- | --- | --- | --- |
| Group 2a | Metformin group | Metformin | Normal diet +150 mg/kg/day | 6 weeks |
| Group 2b | *SFEx* treatment group | *SFEx* | Normal diet +150 mg/kg/day | 6 weeks |
| Group 2c | *PPEx* treatment group | *PPEx* | Normal diet +150 mg/kg/day | 6 weeks |
| Group 2d | *ALEx* treatment group | *ALEx* | Normal diet +150 mg/kg/day | 6 weeks |
| Group 2e | SFPFr treatment group | *SFPFr* | Normal diet +150 mg/kg/day | 6 weeks |
| Group 2f | STZ group | No treatment | Normal diet | 6 weeks |

**Supplementary Table 4:** Grid center coordinates and grid box dimensions chosen for autodocking in PyRx.

| Enzymes with PDB IDs | Grid center x y z coordinates | Grid box dimensions |
| --- | --- | --- |
| Salivary amylase 1SMD | x= 8.3486, y= 58.7042, z= 19.0956 | x= 55.9308, y= 72.0793, z= 56.4713 |
| DPPIV 1NU6 | x= 56.8377, y= 61.7692, z= 52.5542 | x= 89.4385, y= 89.2338, z=126.4175 |
